# Identification of energy metabolism-related biomarkers for risk prediction of heart failure patients using random forest algorithm

**DOI:** 10.3389/fcvm.2022.993142

**Published:** 2022-10-11

**Authors:** Hao Chen, Rui Jiang, Wentao Huang, Kequan Chen, Ruijie Zeng, Huihuan Wu, Qi Yang, Kehang Guo, Jingwei Li, Rui Wei, Songyan Liao, Hung-Fat Tse, Weihong Sha, Zewei Zhuo

**Affiliations:** ^1^Department of Gastroenterology, Guangdong Provincial People's Hospital, Guangdong Academy of Medical Sciences, Guangzhou, China; ^2^School of Medicine, South China University of Technology, Guangzhou, China; ^3^The Second School of Clinical Medicine, Southern Medical University, Guangzhou, China; ^4^Department of Gastroenterology, The First Affiliated Hospital of Guangzhou Medical University, Guangzhou, China; ^5^Cardiology Division, Department of Medicine, Queen Mary Hospital, The University of Hong Kong, Hong Kong, Hong Kong SAR, China

**Keywords:** heart failure, energy metabolism, random forest, nomogram, biomarker

## Abstract

**Objective:**

Energy metabolism plays a crucial role in the improvement of heart dysfunction as well as the development of heart failure (HF). The current study is designed to identify energy metabolism-related diagnostic biomarkers for predicting the risk of HF due to myocardial infarction.

**Methods:**

Transcriptome sequencing data of HF patients and non-heart failure (NF) people (GSE66360 and GSE59867) were obtained from gene expression omnibus (GEO) database. Energy metabolism-related differentially expressed genes (DEGs) were screened between HF and NF samples. The subtyping consistency analysis was performed to enable the samples to be grouped. The immune infiltration level among subtypes was assessed by single sample gene set enrichment analysis (ssGSEA). Random forest algorithm (RF) and support vector machine (SVM) were applied to identify diagnostic biomarkers, and the receiver operating characteristic curves (ROC) was plotted to validate the accuracy. Predictive nomogram was constructed and validated based on the result of the RF. Drug screening and gene-miRNA network were analyzed to predict the energy metabolism-related drugs and potential molecular mechanism.

**Results:**

A total of 22 energy metabolism-related DEGs were identified between HF and NF patients. The clustering analysis showed that HF patients could be classified into two subtypes based on the energy metabolism-related genes, and functional analyses demonstrated that the identified DEGs among two clusters were mainly involved in immune response regulating signaling pathway and lipid and atherosclerosis. ssGSEA analysis revealed that there were significant differences in the infiltration levels of immune cells between two subtypes of HF patients. Random-forest and support vector machine algorithm eventually identified ten diagnostic markers (MEF2D, RXRA, PPARA, FOXO1, PPARD, PPP3CB, MAPK14, CREB1, MEF2A, PRMT1) for risk prediction of HF patients, and the proposed nomogram resulted in good predictive performance (GSE66360, AUC = 0.91; GSE59867, AUC = 0.84) and the clinical usefulness in HF patients. More importantly, 10 drugs and 15 miRNA were predicted as drug target and hub miRNA that associated with energy metabolism-related genes, providing further information on clinical HF treatment.

**Conclusion:**

This study identified ten energy metabolism-related diagnostic markers using random forest algorithm, which may help optimize risk stratification and clinical treatment in HF patients.

## Introduction

Heart Failure (HF) is a complex ailment that characterized by multidimensional nature and primarily results from myocardial infarction, cardiomyopathy, abnormal cardiac load, and arrhythmias. The morbidity and mortality of HF have increased rapidly in recent years, particularly among the elderly (1), and a majority of hospitalized patients die within 5 years of admission (2), affecting over 64 million patients' quality of life (3, 4). The first step in improving the clinical management and survival rate of patients with HF is the rapid and accurate diagnosis of the disease. The current clinical diagnosis of HF relies on a spectrum of biochemical markers, including BNP and NT-proBNP (5, 6). However, several researches demonstrated that BNP lacks sensitivity and specificity as they could increase in various non-HF diseases such as pulmonary arterial hypertension and renal failure (7). Furthermore, BNP and echocardiography is operator-dependent, which limits its diagnostic precision to some degree (5, 8). The emergence of gene testing raised hope for early and diagnosis of HF, which helps better understand the mechanisms underlying the development of HF and identify the potential diagnostic markers. Several biomarkers have been identified to serve as diagnostic and prognostic markers for HF patients (9), whereas the ability of individual markers to differentiate between disease and healthy controls is usually not very powerful (10). Therefore, searching for novel multi-biomarker diagnostic profile was urgently needed to more accurate diagnosis and develop new therapeutic targets in HF patients.

Perturbations of cardiac energy metabolism is an important characteristic of heart failure in the early stages (11–13). During aerobic conditions, the healthy heart derives its contractile energy from fatty acids and glucose, whereas this balance is disrupted under cardiac stress condition, which can have a profound impact on the function of the heart (14). The current study has indicated that energy metabolism is promoted in early and compensated HF states and that a decrease in metabolic capacity may result in the progressive defects seen in more severe cases of HF (10). It is likely that targeted improvements in energy metabolic efficacy will improve the symptomatic status of HF patients. For example, some energy metabolism-related genes such as UCP2 and PRMT1 were found to modulate the energy metabolism in cardiomyocytes after HF, which involved in remodeling of the ventricular wall and the maintenance of cardiac function (15, 16). More importantly, some energy metabolism-related genes like the myocyte enhancer factor 2 (MEF2) family, including MEF2A, MEF2D, has been considered as core transcription factors in cardiac development and reprogramming (17), which may serve as a potential candidate gene for the cardiac abnormalities (18). These studies suggest that further understanding of the value of the energy metabolism in HF patients will be crucial to clarifying the process of HF and developing new therapeutic options (19).

Given the huge burden of HF and the important role of energy metabolism, we obtained energy metabolism-relate DEGs between HF and non-heart failure (NF) samples in the GEO database. By the help of these DEGs data, we used random forest algorithm (RF) and support vector machine (SVM) to identify the diagnostic biomarkers in HF and constructed and validated an energy metabolism-related genetic diagnostic nomogram to predict the risk of HF. Moreover, we predicted key genes related drugs and miRNA. In a word, this study could provide theoretical support for early warning signs of HF and assist in improving risk stratification and guiding clinical decision-making.

## Materials and methods

### Data collection

The two profiling datasets, GSE66360 (*n* = 99) (20) and GSE59867 (*n* = 436) (21) were obtained from gene expression omnibus database (GEO, http://www.ncbi.nlm.nih.gov/geo/). The training group (GSE66360) includes HF patients induced by acute myocardial infarction (*n* = 49) and non-HF cohort (*n* = 50), and the test group (GSE59867) have HF (*n* = 34) and non-HF (*n* = 30). Normalization of the microarray data was performed using the normalize quantiles function of the preprocessCore package in R software (version 3.4.1). The probes were transformed into gene symbols based on the annotation information provided in the platform. These datasets were stripped of probes corresponding to multiple genes, and then we calculated the average expression value of each gene measured by multiple probes as the final expression value. The energy metabolism-related genes were obtained through the Molecular Signatures Database (http://www.gsea-msigdb.org/gsea/msigdb/) that is one of the most widely used and comprehensive databases of gene sets for performing gene set enrichment analysis. In total, 48 genes related to energy metabolism were collected from Wiki Pathways.

### Differential expression analysis of energy metabolism-related genes

The gene expression profiling was annotated through the corresponding annotation packages of the R software. The “limma” R software package was applied to analyze the differential expressed energy metabolism-related genes. Those genes which met the criteria (*P* < 0.05 and |log2(fold change)|>1) were considered as DEGs. The heatmap and boxplot were constructed to show the differential expressed genes which were highlighted. Finally, the chromosomal locations of the DEGs were demonstrated using Circos and the protein-protein interaction (PPI) networks of the DEGs were predicted by the search tool for the retrieval of interacting genes (STRING, version 11.5; https://cn.string-db.org) with minimum required interaction score ≥ 0.7 (22).

### Consensus clustering for heart failure samples

According to energy metabolism-related genes, HF samples were grouped into different classifications using “Consensus Cluster Plus” package in R software (23). Based on the consensus matrix (CM) and cumulative distribution function (CDF) curves of the consensus score, we determined the optimal cluster number (24). Meanwhile, we performed principal component analysis (PCA) between clusters. The “limma” package was then applied to screen for the energy metabolism-related genes between clusters (|log_2_(fold change)|>2 and *P* < 0.05).

### Functional and pathway enrichment analysis

To assess the functional enrichment of DEGs identified between clusters, we performed Gene Ontology (GO) and Kyoto Encyclopedia of Genes and Genomes (KEGG) pathways enrichment analyses using “ClusterProfiler” R package (25). GO database describes our understanding of biology from three GO domains, including biological process (BP), molecular function (MF), and cellular component (CC). The KEGG database provides information about high-level functions in the biological system.

### Immune landscape analysis

ssGSEA is an extension method of the Gene set enrichment analysis analysis (GSEA), using for quantifying infiltrating immune cells. This tool allows the definition of an enrichment score that represents the absolute enrichment level of the gene sets in each sample within a given dataset. To investigate the association between immune infiltration level and two subtypes, ssGSEA was performed by the R package “GSVA” to investigate the differences in immune cell infiltration in two clusters (26). Person method was used to calculate the correlation between immune cells and genes related to energy metabolism.

### Identification of energy metabolism-related diagnostic biomarkers

To screen significant diagnostic biomarkers in HF, two machine learning algorithms including RF and SVM were performed on the identified energy metabolism-related DEGs. These two models were analyzed using the explanatory feature of the R package “DALEX.” Optimal models were chosen by plotting residual distributions. The receiver operating characteristic (ROC) curve was used to assess the diagnostic performance of two models (6). The area under the curve (AUC) was used to measure the overall predictive validity of the risk in HF where AUC = 0.50 signals random prediction, 0.60 < AUC ≤ 0.70 signals poor, 0.70 < AUC ≤ 0.80 signals fair, 0.80 < AUC ≤ 0.90 signals good and AUC > 0.90 signals excellent validity. Based on out-of-band data, we calculated the average modeling error rate for all genes using the R package “random forest.” A random forest model was then constructed, and the Gini coefficient method was used to calculate dimensional importance value (7).

### Construction and validation of the nomogram

Based on the identified energy metabolism-related diagnostic biomarkers by random forest algorithm, we further established a nomogram to predict the occurrence of HF using R package “rms.” The “Points” column indicates the score for each gene below, and the “Total Points” column represents the sum of all the scores. Specifically, we obtained the “Points” for each gene by drawing a line straight upward from each gene to the point scale in the nomogram. The “Points” were then added together and positioned on the scale of “Total points” to further convert them into risk probability of HF. The calibration curve was used to assess the nomogram's predictive accuracy, while the ROC curve was used to examine the predictive power of the model.

### Prediction of biomarker-related miRNA and drugs

“Enrichr” database is a comprehensive online tool for gene enrichment analysis, including a large number of genomic annotation libraries that can be used for analysis and download, such as transcription, pathways, ontology (GO), diseases/drugs, cell types, which may accumulate biological knowledge for further biological discoveries. In this manuscript, we predicted the regulatory correlation between miRNA with 10 diagnostic biomarkers in “Enrichr” database (https://maayanlab.cloud/Enrichr/) and visualized it using Cytoscape 3.9.1 software. Meanwhile, the related drugs were also evaluated in Enrichr.

### Statistical analysis

Statistical analysis was performed by R (version 4.1.1) software. Perl and “limma” package were used to analyze the data. Using “Consensus Cluster Plus” package to classify the samples. *T*-test or Wilcoxon rank-sum test were applied to analyze the continuous variables according to the normality. Pearson chi-square test was applied to examine the differences of categorical variables. All significant thresholds were set at a two-sided *P* < 0.05.

## Results

### Identification of differentially expressed energy metabolism-related genes

**Figure 1:**
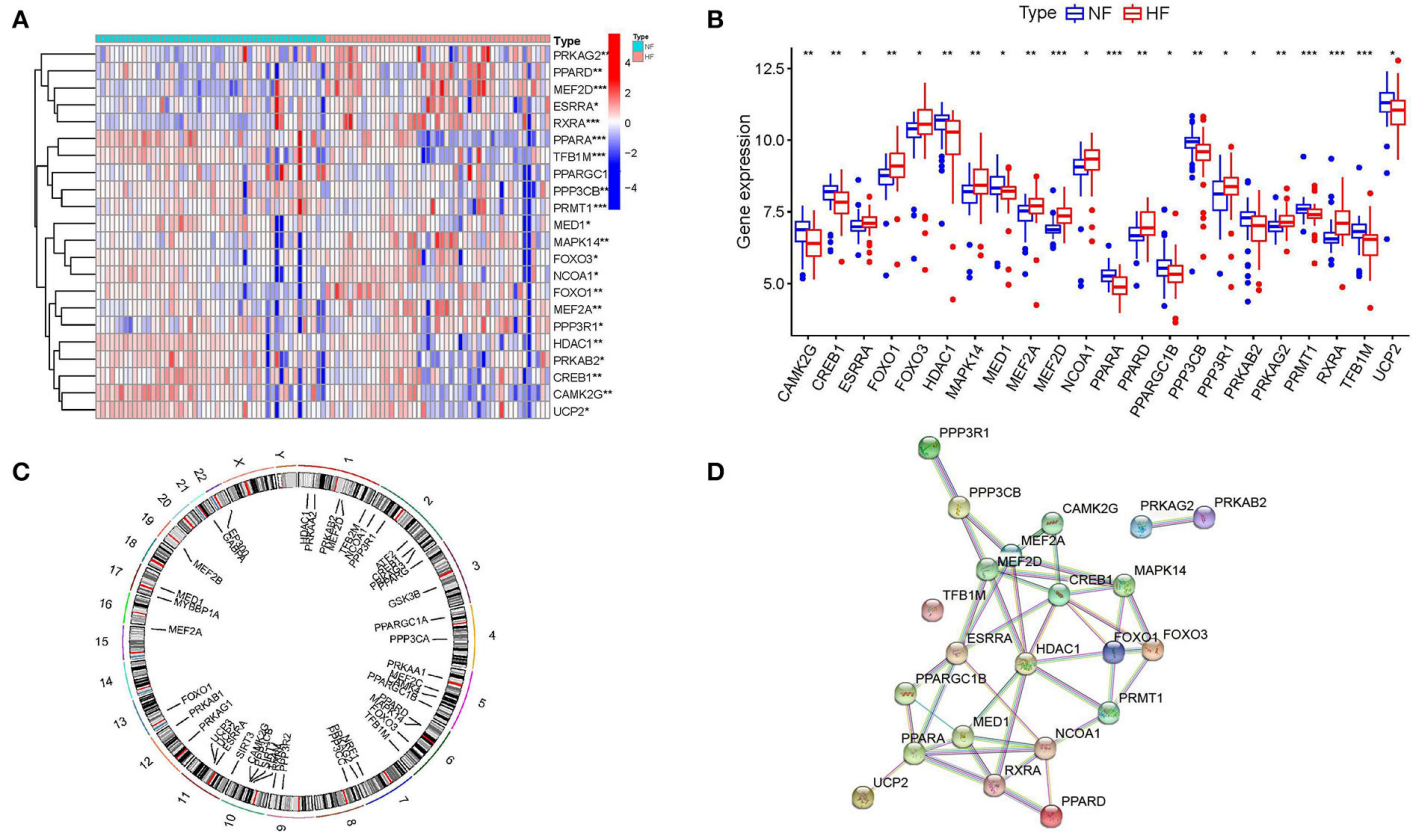
Identification of differentially expressed energy metabolism-related genes. **(A,B)** Expressions of differentially expressed energy metabolism-related genes. **(C)** Circos plot showing the location of genes in 22 chromosomes. **(D)** Protein-protein interaction networks.

A total of 22 energy metabolism-related DEGs, including PRKAG2, PPARD, MEF2D, ESRRA, RXRA, PPARA, TFB1M, PPARGC1B, PPP3CB, PRMT1, MED1, MAPK14, FOXO3, NCOA1, FOXO1, MEF2A, PPP3R1, HDAC1, PRKAB2, CREB1, CAMK2G, UCP2, were identified as different expression genes (Figure 1A). Among them, 11 genes were significantly downregulated and 11 genes were significantly upregulated (Figure 1B). A gene's chromosomal location can provide information about its evolutionary history, including gene duplication patterns, and gene duplication events (27). Herein, chromosomal location information of these energy metabolism-related genes was performed by the genome visualization tool named CIRCOS, providing insights into the evolution of these gene family. We can find that a total of 44 energy metabolism-related genes were distributed throughout the 18 chromosomes and the highest numbers of these genes (*n* = 6) were located on chromosome 2 (Figure 1C). PPI analysis was conducted to explore the interactions of these energy metabolism-related DEGs at the protein level. As shown in Figure 1D, the PPI network showed that MEF2A, MEF2D, CREB1, HDAC1, MED1 and ESRRA may have higher numbers of interacted proteins.

### Molecular subtype of heart failure based on energy metabolism-related genes

**Figure 2:**
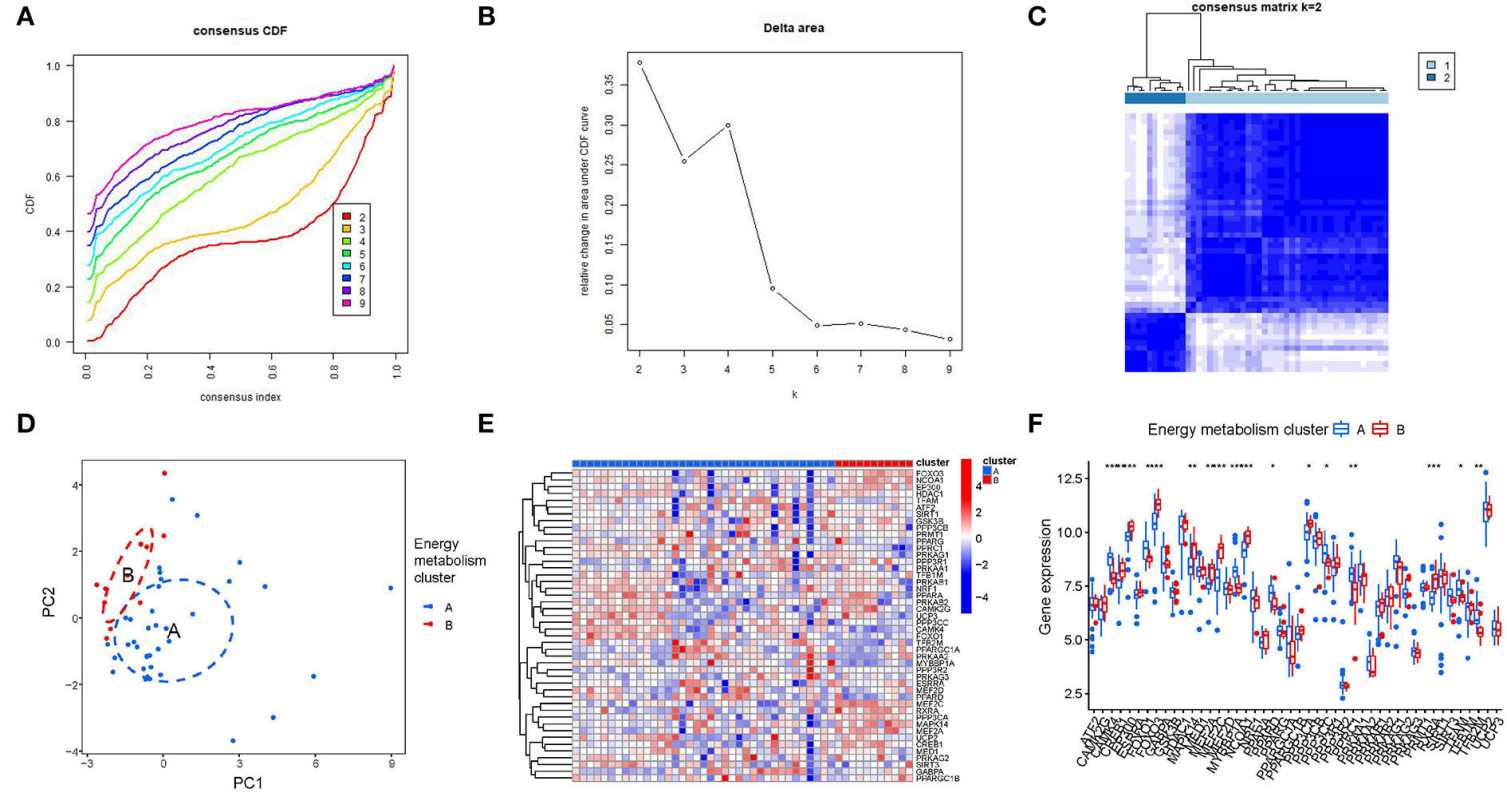
Consensus clustering analysis of energy metabolism-related genes. **(A)** The cumulative distribution function (CDF) curve of samples in the HF cohort. **(B)** The relative change in area under the CDF curve for k = 2–9. **(C)** Sample clustering heatmap when consumption k = 2. **(D)** PCA analysis for cluster A and cluster B. **(E,F)** The different expression of 48 genes between two clusters.

Consensus clustering is a method that provides quantitative analysis results to determine possible subtypes based on gene expression profiling data, which can be used to discover new molecular subtypes and thus redefine disease classification. In this study, consensus clustering was performed to identify HF subtypes based on energy metabolism-related genes. According to the CDF curve and the CDF Delta area curve, clustering results are relatively stable when the number of clusters was set to 2 (Figures 2A,B). Figure 2C presented a heatmap of clustering results (k = 2) and the PCA result showed a clear distribution between cluster A and cluster B (Figure 2D), suggesting that energy metabolism-related genes have potential diagnostic value for HF patients. Figure 2E presented a heatmap of 48 genes expression level between two clusters. The result of the boxplot showed that FOXO1, RXRA, CREB1, MAPK14, MEF2A,PPARD, FOXO3, EP300, CAMK3, MEF2C, MYBBP1A, NCOA1, PPP3CA, PPP3CC, PPRC1, TFAM, TFB2M were significant differential expressed between cluster A and cluster B (*P* < 0.05, Figure 2F).

### Functional analyses of different clusters of HF patients

**Figure 3:**
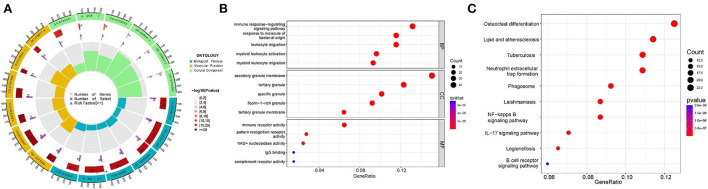
Functional analyses of DEGs identified between clusters. **(A,B)** Gene Ontology (GO) functional analysis. **(C)** Kyoto Encyclopedia of Genes and Genomes (KEGG) pathway enrichment analysis.

To examining the differences in gene functions and pathways between the subgroups grouped by energy metabolism-related genes, we extracted DEGs using the “limma” R package with a threshold of FDR < 0.05 and |log_2_FC | ≥ 2. A total of 378 DEGs were identified between cluster A and cluster B, and then GO and KEGG enrichment analysis were conducted on these DEGs. The result demonstrated that the DEGs were mainly correlated with immune response regulating signaling pathway (BP), secretory granule membrane (CC), immune receptor activity (MF) (Figures 3A,B, Supplementary Figure 1), osteoclast differentiation, and lipid and atherosclerosis (Figure 3C).

### Assessment of the immune infiltration level between clusters

**Figure 4:**
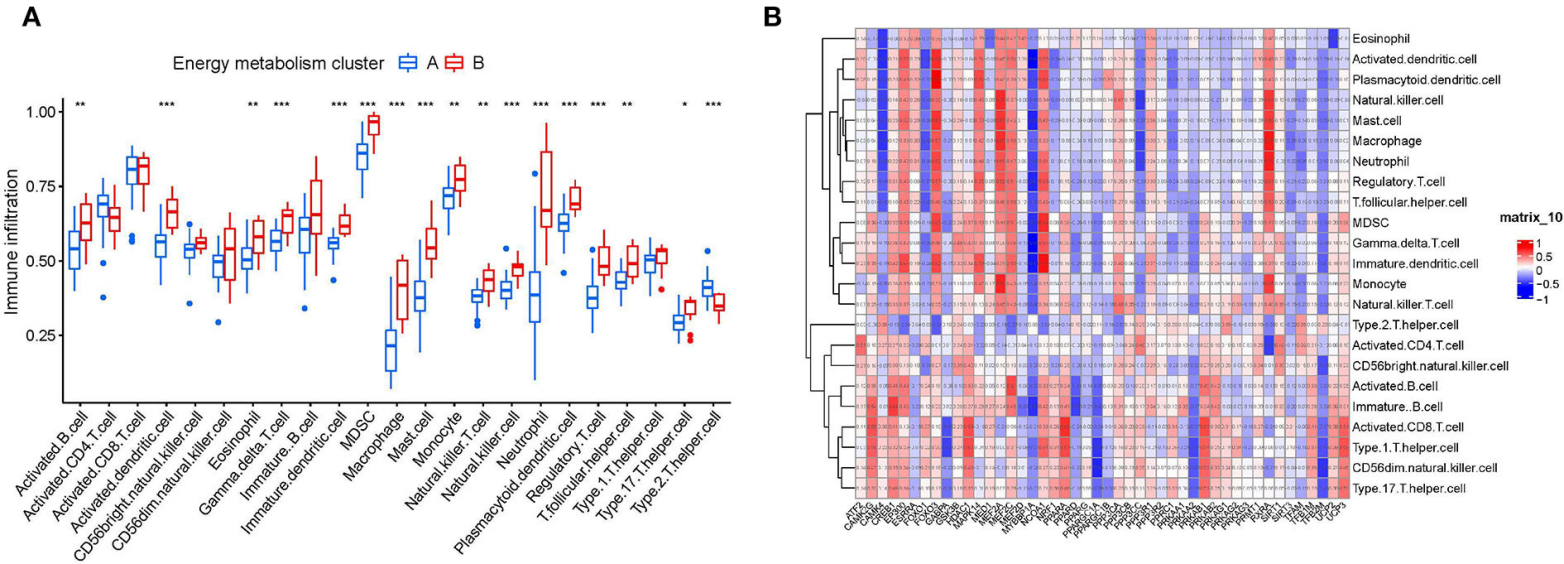
Correlation analysis between energy metabolism-related genes and immune microenvironment. **(A)** The relationship between two clusters and the level of immune cell infiltration. *p-value < 0.05; **p-value < 0.01; ***p-value < 0.001; ****p-value < 0.0001. **(B)** The relationship between 46 energy metabolism-related genes and immune cell infiltration.

Based on the results of functional analyses, we further explore the correlation of immune cell infiltration level between two clusters. First, we confirmed the role of immune cell infiltration in HF development. As the Supplementary Figure 1 shown, nearly half of immune cell levels (12/23) were found more abundant in HF samples vs. the healthy control (*P* < 0.001), suggesting that patients with HF already show signs of systemic-immune activation, and may contribute to the progression to HF. More importantly, we found that most of immune cells (17/23) have also different infiltration levels in two clusters (Figure 4A). Compared to the cluster B, cluster A generally had lower levels of immune cell infiltration, especially of activated dendritic cell, gamma delta T cell, immature dendritic cell, MDSC, macrophage, mast cell, natural killer cell, neutrophil, plasmacytoid dendritic cell, regulatory T cell, and Type 2 T helper cell (*P* < 0.001). Furthermore, we assessed the correlation between 48 energy metabolism-related genes and 24 immune cells (Figure 4B). There was a strong correlation between the expressions of most of the energy metabolism-related genes and the infiltration of immune cells, especially MAPK14, FOXO1, and RXRA.

### Establishment and evaluation of RF and SVM model

**Figure 5:**
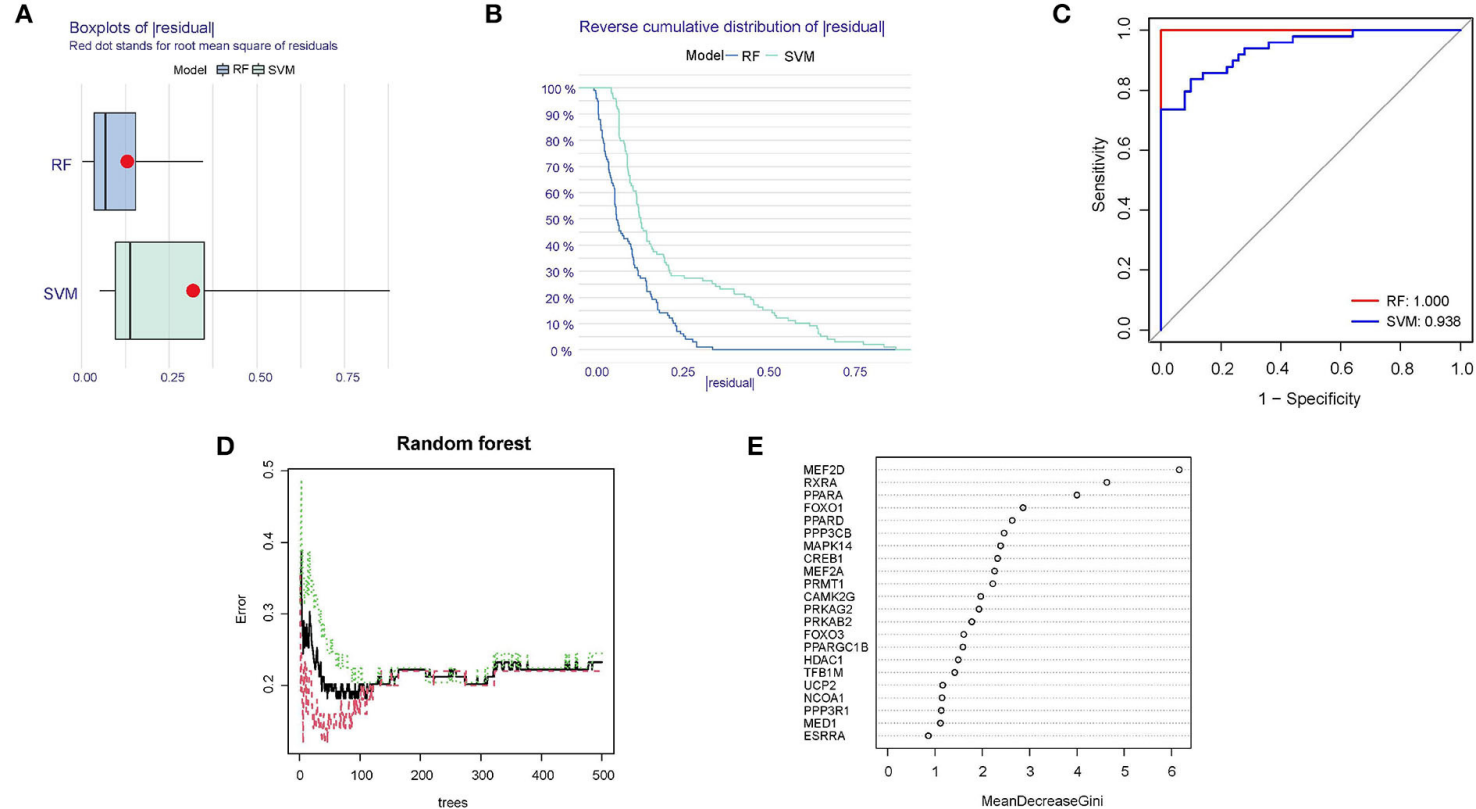
Identification of diagnostic biomarkers using RF and SVM model. **(A)** Boxplots of the residuals of the sample. Red dot stands for root mean square of residuals. **(B)** Cumulative residual distribution map of the sample. **(C)** AUC verification results of the two models on the training dataset. **(D)** The influence of the number of decision trees on the error rate. **(E)** Results of the Gini coefficient method in the random forest classifier.

Compared RF and SVM, according to the training dataset (GSE66360), we found that RF had the less sample residual (Figures 5A,B). Similarly, the AUC of the random forests model (AUC = 1.000) and SVM model (0.938) showed that RF model had a higher degree of differentiation (Figure 5C). As shown in Figure 5D, 400 decision trees were selected as the final model parameter based on the relationship plot between the model error and the number of trees. Figure 5E presented the variable importance of the output results in the process of the construction of random forest model based on the Gini coefficient method. For further analysis, 10 genes with a significance > 2 were identified as candidate genes. Among ten variables, MEF2D, RXRA, and PPARA were the most important, followed by FOXO1, PPARD, PPP3CB, MAPK14, CREB1, MEF2A, PRMT1.

### Establishment of the clinical nomogram

**Figure 6:**
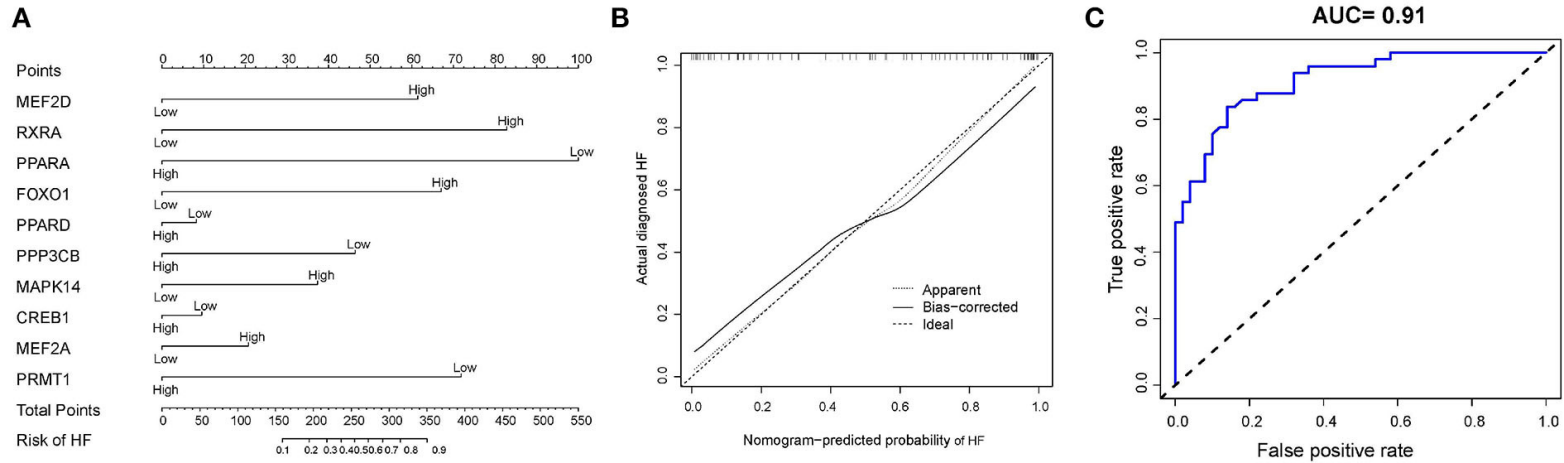
Construction of a nomogram model for HF diagnosis in training cohort (GSE66360). **(A)** The nomogram was used to predict the occurrence of HF. **(B)** Calibration curve to assess the predictive power of the nomogram model. **(C)** The receiver operating characteristic (ROC) analysis of nomogram.

**Figure 7:**
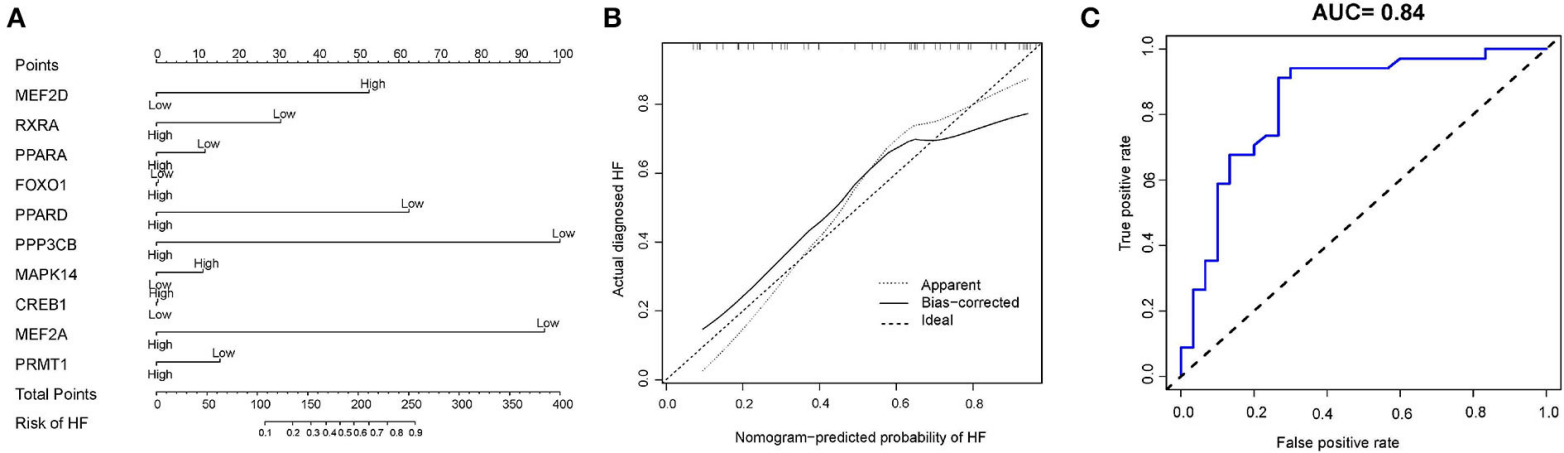
Construction of a nomogram model for HF diagnosis in test cohort (GSE59867). **(A)** The nomogram was used to predict the occurrence of HF. **(B)** Calibration curve to assess the predictive power of the nomogram model. **(C)** The receiver operating characteristic (ROC) analysis of nomogram.

Based on the ten diagnostic biomarkers from the RF model, we developed a clinical predictive nomogram (Figure 6A) and people can use the nomogram score to predict the risk of HF. Using calibration curve, we evaluated the predictive accuracy of the nomogram and the result indicated the nomogram has high accuracy for risk prediction of HF (Figure 6B). The corresponding ROC analysis revealed that the AUC value of the constructed diagnostic model was 0.91, which proved the predictive performance of this clinical nomogram (Figure 6C). We next verified the stable of the nomogram using the test cohort (GSE59867) (Figure 7A). The calibration curve of this nomogram was close to diagonal line (Figure 7B) and the AUC value was 0.84 (Figure 7C), further validating the accuracy and robustness of our nomogram.

### Prediction of related drugs and miRNA

**Figure 8:**
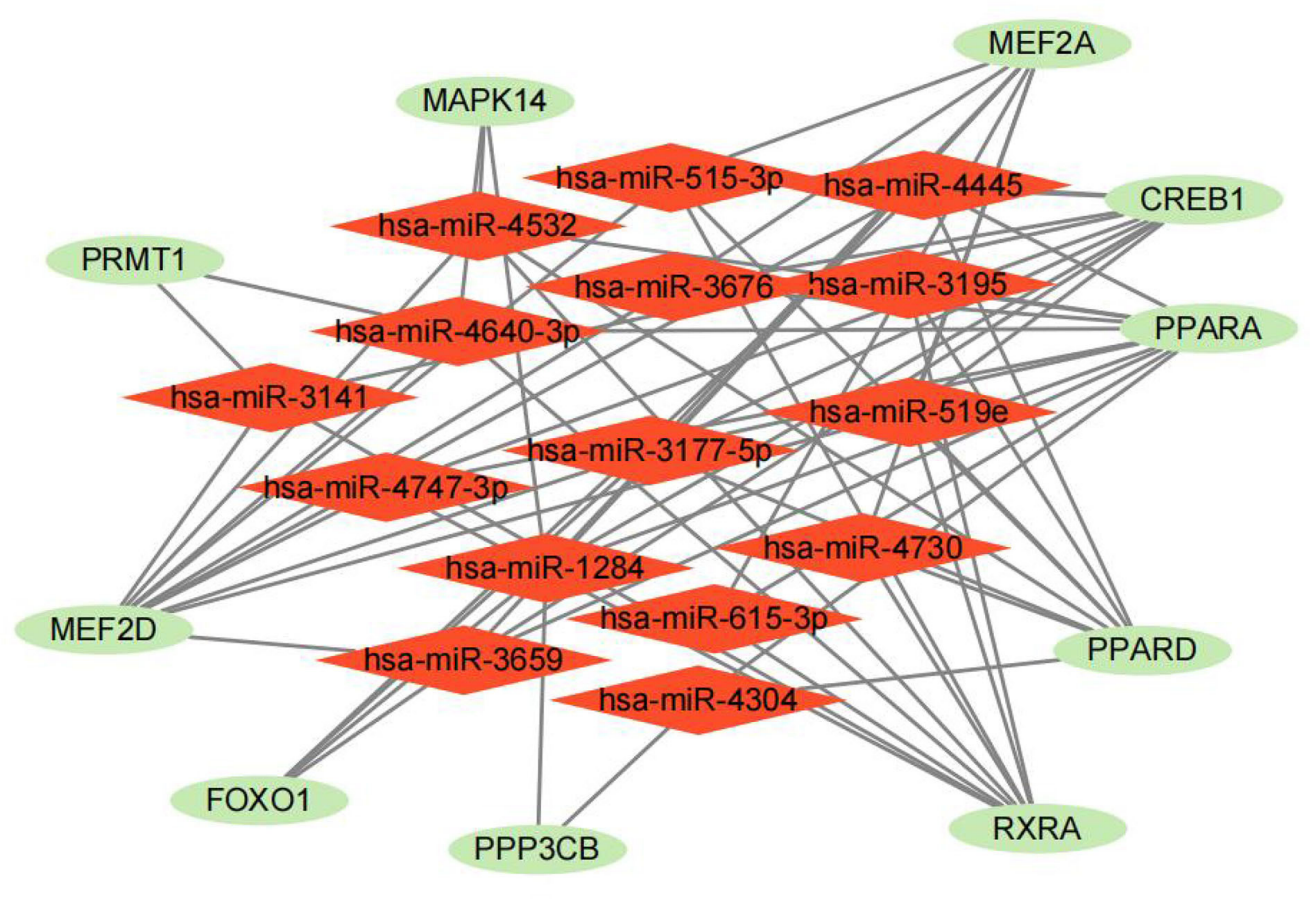
The prediction of energy metabolism-related miRNA.

Ten drugs were identified the energy metabolism-related drugs, which may be the potential therapies for heart failure target (Table 1). RXRA, PPARA and PPARD were the top three genes most relevant to these drugs, indicating that these biomarkers have great potential to serve as a drug target for HF treatment. Furthermore, we constructed a miRNA–gene association network and displayed 15 potential miRNA targets of 10 energy metabolism-related biomarkers, which may play a regulatory role in the development of HF (Figure 8). Among them, miR-3177-5p and miR-1284 contributed to the regulation of the highest number of target genes (*n* = 6), followed by miR-4532, miR-4640-3p, miR-4445, miR-515-3p, miR-519e, and miR-3659 (*n* = 5).

**Table 1:** The prediction of energy metabolism-related drugs.

**Index**	**Name**	***P*-value**	**Adjusted**	**Odds**	**Combined**	**Gene**
			***P*-value**	**ratio**	**score**	
1	phthalic acid CTD 00001559	5.02E-08	2.15E-05	658.58	11068.58	RXRA;PPARA;PPARD
2	DIETHYL PHTHALATE CTD 00000348	1.02E-07	2.91E-05	503.52	8105.11	RXRA;PPARA;PPARD
3	Pirinixic acid TTD 00010254	1.23E-05	3.30E-04	555.03	6273.06	RXRA;PPARA
4	Difenoconazole CTD 00003609	1.48E-05	3.84E-04	499.5	5554.53	RXRA;PPARD
5	15(R)-Prostaglandin D2 CTD 00007048	1.75E-05	4.16E-04	454.07	4973.59	PPARA;PPARD
6	Triphenyltin hydroxide CTD 00000355	1.75E-05	4.16E-04	454.07	4973.59	RXRA;PPARD
7	4602-84-0 CTD 00005951	2.04E-05	4.57E-04	416.21	4494.85	RXRA;PPARA
8	gemfibrozil CTD 00007055	4.87E-07	6.91E-05	285.14	4144.43	RXRA;PPARA;PPARD
9	7614-21-3 CTD 00000893	2.35E-05	4.57E-04	384.17	4094.01	RXRA;PPARA
10	gemfibrozil TTD 00008191	2.35E-05	4.57E-04	384.17	4094.01	RXRA;PPARA

## Discussion

Heart failure is a deadly chronic disease that owns a high symptom burden and a poor health status. Patients with HF often fail to benefit from treatment as a result of a lack of an early diagnosis, resulting in poor prognosis. It has been demonstrated in numerous studies that HF is caused by severe energy metabolism disorders, resulting in insufficient energy supply to the heart (14). As a consequence, researchers are looking for novel diagnostic biomarkers and investigating the molecular level of energy metabolism in HF, which could lead to a number of positive effects on the clinical outcome of HF. Growing evidence demonstrated that microRNAs and mRNAs may be promising biomarkers of cardiovascular disease and HF in particular (9). However, few studies have examined the aberrantly expressed genes associated with energy metabolism in HF vs. normal tissues. Thus, this study sought to identify candidate biomarkers for detecting HF and explore how energy metabolism may contribute to it.

As far as our knowledge goes, this is a novel study that analyzed GEO datasets to identify diagnostic biomarkers associated with energy metabolism in patients with HF. A total of 22 energy metabolism-related DEGs were identified between HF and normal tissues, including 11 upregulated genes and 11 downregulated genes. The result of consensus clustering analysis indicated that HF patients could be classified into two clusters based on 48 energy metabolism-related genes. Generally speaking, proposing new subtypes through the clustering could contribute to provide more precise treatment options (28) and thus our finding is the first time to prove potential diagnostic and therapeutic utility of energy metabolism gene set in HF. GO and KEGG enrichment analyses indicated that DEGs between the two clusters are mainly associated with the immune response regulating signaling pathway. Based on the result of these functional analyses, we further explored the association of immune infiltration between two clusters and found that HF patients have different immunity status across different cluster. These results generally agree with the previous finding that immune activation plays an essential role in the progression of HF (29). In fact, various forms of HF may be affected by the immune system, according to more recent evidence (30, 31). For example, regulatory T cell may be crucial in suppressing cellular immune responses, controlling both inflammation and infection during the development of HF (32). Some studies also indicated that inflammation-related effector cytokines, such as IL-17 family members and IL-22 are associated with Th17 cells (33), and these effector cytokines were demonstrated to regulate the MMP/TIMP system to influence myocardial fibrosis (34, 35). Excessive numbers of monocytes, macrophages, dendritic cells, and lymphocytes have been found to increase myocyte apoptosis, hypertrophy, and interstitial fibrosis during chronic heart failure (36). These findings are consistent with our own, demonstrating the validity of the results in the present study as well as the crucial role played by the immune response in HF (37). Thus, it is crucial to precisely control various types of immune cells to ensure a safe and effective treatment for HF patients. Furthermore, more research is required to better understand the role of immune cells in the heart in homeostasis and energy metabolism, aiming to identify the therapeutic methods targeting immune in patients with diverse kinds of HF.

Lacking sensitivity and specificity is the main limitation for the early diagnosis in HF (38–40), whereas novel multi-gene diagnostic biomarker may resolve this dilemma. In our study, 10 energy metabolism-related diagnostic markers were identified using random forest algorithm, which allows diagnosis of HF with high stability and accuracy. Among them, MEF2A and MEF2D, are both essential regulator of cardiac morphogenesis and myogenesis, which can bind specifically to the MEF2 element in the regulatory region of many muscle-specific genes (41). PPARA and PPARD, which mainly regulate fatty acids and lipid metabolism, function as transcription activator for cardiac fatty acid oxidation (42). Similarly, FOXO1 (31–34) and MAPK14 (35, 43) are highly expressed during the progress of cardiac hypertrophy which contributes to HF development. Our immune cell association analysis also noted that MAPK14, FOXO1 have a strong association with immune cells, indicated that these two genes may play immune-inhibiting roles in HF. In the contrast, activated CREB1 may reduce the excessive burden on the heart and heart hypertrophy (44, 45). Loss of PRMT1 in cardiomyocytes causes multifunctional CaMKII dysregulation, resulting in dilated cardiomyopathy and heart failure (15, 46). RXRA, receptor for retinoic acid, is demonstrated to be involved in the adipogenic/lipogenic regulation (47, 48), which has significant correlation with cardiovascular aging process, which contributes to the development of HF phenotype and outcome (49). PPP3CB, belonging to α-catalytic subunit gene family members (50), has been reported to be significantly up-regulated in the atrial myocyte hypertrophy of mitral regurgitation patients (51). Based on above 10 biomarkers, we constructed and validated a novel diagnose nomogram to risk prediction of HF patients. Compared with other predicted nomogram (AUC = 0.655~0.720) (52), the AUC value of our nomogram, which was 0.91 in the training cohort (GSE66360) and 0.84 in the test cohort (GSE59867), indicated that it may have exceptional potential for making an early diagnosis of HF from patient blood samples.

In addition to the construction of clinical nomogram, anticipating gene-miRNA and gene-drug interactions is also an important task that helps understand the potential miRNA targets of energy metabolism-related genes and better guide clinical medication in HF. In this study, we predicted 10 related drugs using Enrichr based on 10 energy metabolism-related biomarkers. Most of these drugs are still in its infancy and remains exploratory. Among them, pirinixic acid, a potent PPARA receptor activator, exhibits anti-inflammatory properties in human neutrophils and may be useful as therapeutic agents (53). In fact, upregulating PPARA has been reported to promote mitochondrial energy metabolism and prevents HF (50) and the activated PPARA could increase high-density lipoprotein and reduce plasma lipids (54). Another predictive drug, diethyl phthalate was found to induce the antioxidant and immune responses in zebrafish embryos under DBP/DEP exposure (48). In a Helsinki Heart Study of primary prevention, gemfibrozil treatment has been found to reduce coronary events by 34% (55), which can also be considered for HF management. However, most predicted drugs lacked clinical outcomes and these components still need further research for clinical targeted therapy. As for miRNA, it is not only a diagnostic biomarker, but also a therapeutic target for HF. In our prediction, we constructed a miRNA-gene network and these targets and miRNA may be served as potential biomarkers in HF. For example, regulating miR-615-3p/HMGB3 axis have been reported to promote glycolysis under hypoxic conditions at least partly. Notably miR-519e had similar mechanism (56), which helps us better understanding of the molecular mechanism of energy metabolism in HF.

Limitation also exists in this study. First, the important clinical information could not be obtained since it was retrospective in our study. Moreover, the functions and molecular mechanisms of these ten biomarkers in HF need to be further studied *in vitro* and *in vivo* experiments. Furthermore, it will be necessary to conduct large-scale prospective studies with strict follow-up protocols in the future to confirm the clinical feasibility of the proposed biomarkers.

## Conclusions

In summary, we applied random forest-based feature selection to identify the 10 high-performance biomarkers for HF classification, and a clinical nomogram was constructed to visualizes the 10 identified biomarkers, which could better guide the clinical decisions. Further prediction potential miRNA and drugs of these 10 biomarkers provided further application on clinical HF treatment. We proved potential diagnostic utility of energy metabolism gene set in HF, and hope to assist in improving risk stratification and provide the potential treatment targets in HF.

## Data availability statement

The datasets presented in this study can be found in online repositories. The names of the repository/repositories and accession number(s) can be found in the article/Supplementary material.

## Author contributions

H-FT, WS, and ZZ designed, revised, and supervised the study. HC, RJ, RZ, and HW analyzed and organized the data. WH, KC, QY, KG, JL, RW, and SL generated the figure and tables. HC, RJ, WH, and ZZ wrote this manuscript. All authors reviewed and approved the final manuscript.

## Funding

This work was funded by the National Natural Science Foundation of China (82171698, 82170561, 81300279, and 81741067), the Natural Science Foundation for Distinguished Young Scholars of Guangdong Province (2021B1515020003), Natural Science Foundation of Guangdong Province (2022A1515012081), the Climbing Program of Introduced Talents and High-level Hospital Construction Project of Guangdong Provincial People's Hospital (DFJH201803, KJ012019099, KJ012021143, and KY012021183).

## Conflict of interest

The authors declare that the research was conducted in the absence of any commercial or financial relationships that could be construed as a potential conflict of interest.

## Publisher's note

All claims expressed in this article are solely those of the authors and do not necessarily represent those of their affiliated organizations, or those of the publisher, the editors and the reviewers. Any product that may be evaluated in this article, or claim that may be made by its manufacturer, is not guaranteed or endorsed by the publisher.

## Supplementary material

Supplementary Figure 1 Boxplot of immune cell subtypes level between HF samples and normal samples. ^*^represents *P* < 0.05, ^**^represents *P* < 0.01, ^***^represents *P* < 0.001.

Supplementary Table 1 Results of GO analysis of DEGs.

## Abbreviations

HF: Heart failure
DEGs: differentially expressed genes
RF: random forest algorithm
SVM: support vector machine
ROC: Receiver operating characteristic
NF: non-heart failure people
GEO: Gene Expression Omnibus
CM: consensus matrix
PCA: Principal component analysis
ssGSEA: Single-sample gene set enrichment analysis
CDF: cumulative distribution function
MEF2A: Myocyte enhancer factor 2A
MEF2D: myocyte enhancer factor 2D
PPARA: Peroxisome proliferator-activated receptor alpha
PPARD: peroxisome proliferator-activated receptor delta
FOXO1: forkhead box O1
MAPK14: mitogen-activated protein kinase 14
CREB1: cAMP responsive element binding protein 1
PRMT1: protein arginine methyltransferase 1
CaMKII: cardiomyocytes causes multifunctional Ca2+/calmodulin-dependent kinase II
RXRA: Retinoid X receptor alpha
PPP3CB: Protein phosphatase 3 catalytic subunit beta
BNP: B-type natriuretic peptide
NT-proBNP: N-terminal pro-B-type natriuretic peptide
CTD: The Comparative Toxicogenomics Database
TTD: The Therapeutic Target Database
UCP2: uncoupling protein 2
PRMT1: protein arginine methyltransferase 1.
