# Supplementary figures and images for: Identification of energy metabolism-related biomarkers for risk prediction of heart failure patients using random forest algorithm
Source: Front Cardiovasc Med. 2022 Oct 11;9:993142. doi: 10.3389/fcvm.2022.993142 (PMC9593065; doi:10.3389/fcvm.2022.993142)

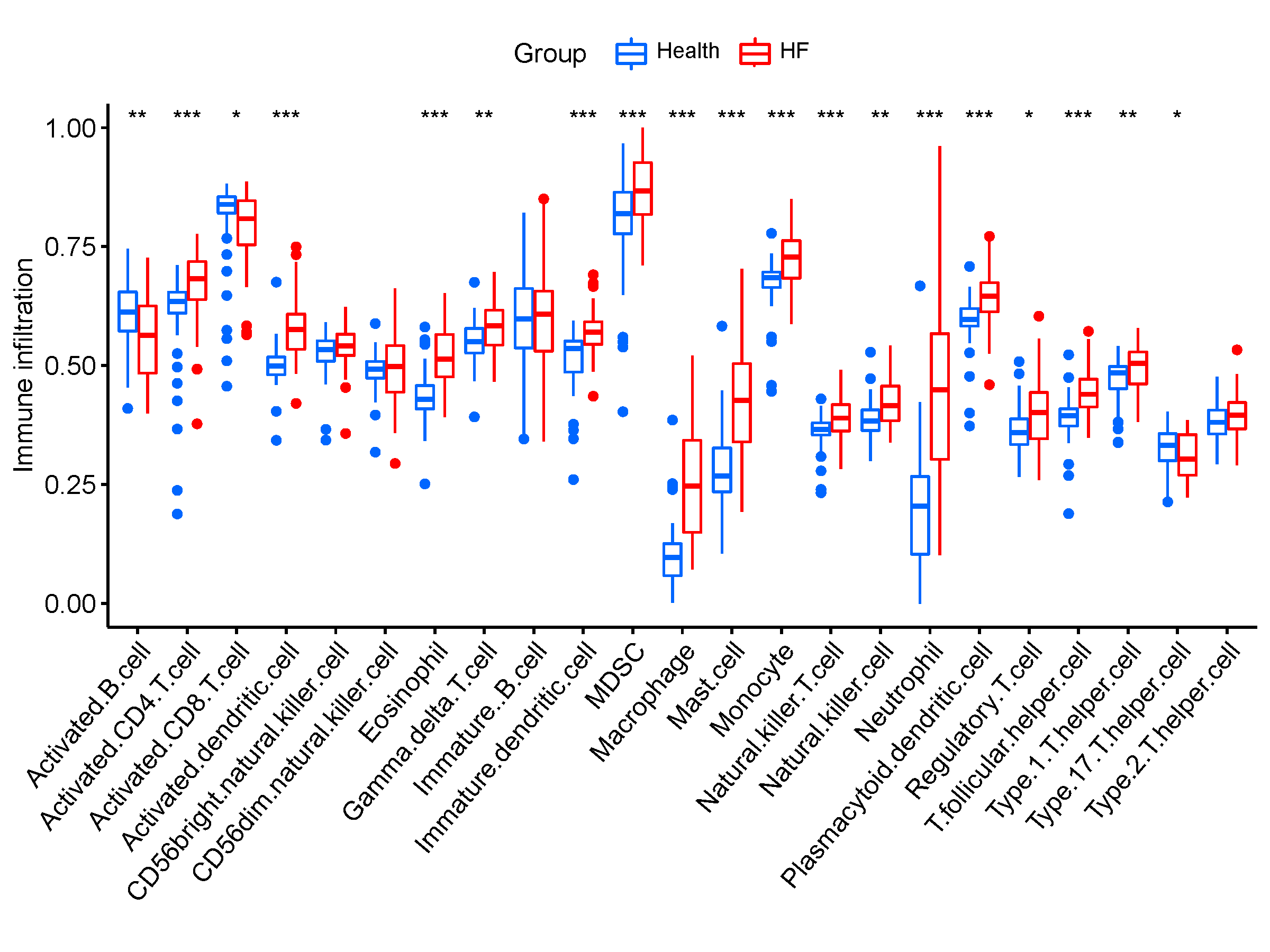

Supplement: Supplementary Figure 1 — Boxplot of immune cell subtypes level between HF samples and normal samples. *represents P < 0.05, **represents P < 0.01, ***represents P < 0.001. [file Image_1.TIFF]
